# Human Mesenchymal Stem Cells Expressing Erythropoietin Enhance Survivability of Retinal Neurons Against Oxidative Stress: An *In Vitro* Study

**DOI:** 10.3389/fncel.2018.00190

**Published:** 2018-07-31

**Authors:** Suet Lee Shirley Ding, Suresh Kumar, Mohammed Safwan Ali Khan, Pooi Ling Mok

**Affiliations:** ^1^Department of Biomedical Science, Faculty of Medicine and Health Sciences, Universiti Putra Malaysia, Seri Kembangan, Malaysia; ^2^Department of Medical Microbiology and Parasitology, Faculty of Medicine and Health Sciences, Universiti Putra Malaysia, Seri Kembangan, Malaysia; ^3^Genetics and Regenerative Medicine Research Centre, Universiti Putra Malaysia, Seri Kembangan, Malaysia; ^4^Department of Pharmacology, College of Pharmacy, Jouf University, Sakaka, Saudi Arabia; ^5^Department of Pharmacology, Anwarul Uloom College of Pharmacy affiliated to Jawaharlal Nehru Technological University-Hyderabad, Hyderabad, India; ^6^Department of Clinical Laboratory Sciences, College of Applied Medical Sciences, Jouf University, Sakaka, Saudi Arabia

**Keywords:** mesenchymal stem cells, erythropoietin, retinal neurons, oxidative stress, Wharton’s jelly, ocular disorders

## Abstract

Retinal degeneration is a prominent feature in ocular disorders. In exploring possible treatments, Mesenchymal Stem Cells (MSCs) have been recognized to yield therapeutic role for retinal degenerative diseases. Studies have also displayed that erythropoietin (EPO) administration into degenerative retina models confers significant neuroprotective actions in limiting pathological cell death. In this study, we aimed to use MSCs to deliver EPO and to evaluate the ability of EPO to rescue retinal neurons from dying upon reactive oxidative stress induction. We derived human MSCs from Wharton’s jelly (hWJMSCs) of the umbilical cord and cells were transduced with lentivirus particles encoding *EPO* and a reporter gene of green fluorescent protein (*GFP*). The supernatants of both transduced and non-transduced cells were collected and used as a pre-conditioning medium for Y79 retinoblastoma cells (retinal neuron cell line) following exposure to glutamate induction. Retinal cells exposed to glutamate showed reduced mitochondrial depolarization and enhanced improvement in cell viability when incubated with pre-conditioned media of transduced cells. Our results established a proof-of-concept that MSCs could be used as a candidate for the delivery of *EPO* therapeutic gene in the treatment of retinal degenerations.

## Introduction

Retinal degeneration is a structural defect acquired in both inherited (Shintani et al., 2009; Daiger et al., 2013; Tomita et al., 2013) and sporadic ocular disorders (Punzo et al., 2012; Sobrin and Seddon, 2014), such as Age-related Macular Degeneration (AMD) and retinitis pigmentosa. Loss of retinal neurons could lead to either fractional or massive loss of visual acuity. To date, there is no clinically translatable antidote for blindness. Existing conventional treatments such as surgical intervention (Iu and Kwok, 2007; Gaudana et al., 2009; Mead et al., 2015) or drug treatments (Iu and Kwok, 2007; Gaudana et al., 2009), are only indicated for patients with early diagnoses to prevent aggravation of the disorder (Sivan et al., 2016).

The use of Mesenchymal Stem Cells (MSCs) have been described as a potential therapeutic approach in treating numerous degenerative disorders in the brain (Castillo-Melendez et al., 2013; Wyse et al., 2014), spinal cord (Johnson et al., 2010), and kidney (Liu et al., 2013; Wang et al., 2015). In retinal disorders, delivery of MSCs was found to improve retinal morphology and function, and delay its degeneration (Kicic et al., 2003; Lund et al., 2009; Guan et al., 2013; Hu et al., 2013; Tzameret et al., 2014; Leow et al., 2015). Ample studies showed that MSCs could secrete restorative extracellular trophic factors that encourage endogenous cellular recovery and replenishment (Ji et al., 2004; Kang et al., 2012; Sohni and Verfaillie, 2013). In addition, post-transplanted MSCs was evidenced to transdifferentiate into retinal neurons (Tomita et al., 2002; Kicic et al., 2003; Arnhold et al., 2006; Castanheira et al., 2008; Tao et al., 2010; González-Garza and Moreno-Cuevas, 2012; Guan et al., 2013; Hu et al., 2013) and retinal pigment epithelium (Vossmerbaeumer et al., 2009; Huang et al., 2012; Guan et al., 2013) in both *in vitro* and *in vivo* studies.

It is noteworthy that a successful transplantation requires not only the capacity of the transplanted cells to engraft (Mok et al., 2013), but also the ability of the cells to survive in the pathological microenvironment (English and Wood, 2013; Mok et al., 2013). Introducing anti-apoptotic proteins, such as erythropoietin (EPO), may thus aid in enhancing both MSCs survivability and engraftment (Lifshitz et al., 2009; Alural et al., 2014; Liu et al., 2015), leading to improvement in the treatment outcomes of retinal degenerative disorders. EPO is a hormonal glycoprotein involved in the formation of red blood cells (Eckardt and Kurtz, 2005). Recently, studies have shown that EPO proteins and its associated receptors are present in the retina (Ghezzi and Brines, 2004; Caprara and Grimm, 2012). We have also previously reviewed the clinical significance of EPO in the management of ocular disorders (Gawad et al., 2009; Guan et al., 2013) through its anti-apoptotic, anti-inflammatory, anti-oxidative and neuroregenerative properties (Garcia-Ramírez et al., 2011; Chang et al., 2013; Chu et al., 2014; Liu et al., 2015; Shirley Ding et al., 2016).

In this study, we aimed to genetically modify MSCs to produce and secrete human EPO protein and to demonstrate the high potential of dual combination of EPO delivered by MSCs to protect retinal neurons from apoptosis in a glutamate-induced human retinoblastoma (Y79) *in vitro* model. The MSCs were derived from human Wharton’s jelly and the *EPO* gene was introduced by lentiviral transduction. Cellular recovery of human retinoblastoma (Y79) subjected to glutamate at a toxic dose was assessed following incubation with supernatants harvested from *EPO*-transduced MSCs. Our data indicates that the *EPO*-transduced MSCs could rescue human retinal neurons from dying upon neurotoxicity induction with glutamate. This may provide supporting evidence for further evaluation in animal studies.

## Materials and Methods

### Culture and Expansion of hWJMSCs

Human Wharton’s Jelly-derived MSCs were obtained from Cryocord Sdn. Bhd. (Cyberjaya, Malaysia). hWJMSCs were expanded at a density of 3000 cells/cm^2^ in a 25-cm^2^ plastic flask containing 5 mL of MSC culture media composing of Dulbecco’s Modified Eagle Medium with nutrient mixture F-12 (DMEM/F12) medium (Gibco; USA) supplemented with 10% Fetal Bovine Serum (FBS; Gibco), 100 units/mL of penicillin (Gibco), and 100 μg/mL of streptomycin (Gibco). The flask was then transferred into a 5% CO_2_ incubator at 37°C and routinely monitored. Upon reaching to 80% cell confluence, the cells were detached by the addition of 0.25% trypsin–EDTA (Gibco) and centrifuged at 200× g for 8 min. Following centrifugation, the cell pellet was re-seeded into sterile flasks at an equal volume of cell density.

### Immunophenotyping of hWJMSCs

Characterization based on immunophenotyping and the bi-potency differentiation potential of cultured MSCs were evaluated from cells of third up to fifth passages. Following cell detachment with 0.25% trypsin–EDTA, hWJMSCs were suspended in phosphate-buffered saline (PBS; Gibco) pH 7.2 containing fluorescein isothiocyanate (FITC), allophycocyanin (APC) or phycoerythrin (PE)-conjugated monoclonal antibodies for 30 min. The antibodies used were CD90, CD73, CD105, CD29, CD44, HLA-ABC, CD34, CD14, CD45, CD80 and CD86, all obtained from BD Biosciences (Canada). Subsequently, the cells were washed twice with 1× PBS and centrifuged for 5 min at 300× *g* prior to flow cytometric analysis. In parallel, unstained and corresponded fluorochrome of non-specific isotype-labeled cells were used as controls. The stained samples were assessed using BD FACSAria III (BD Biosciences). Gating at FACS acquisition was drawn to exclude any cell death and cell debris. Ten thousand events were acquired and the data from stained cells were acquired using FACSDiva 6.1.3 software (BD Biosciences).

Concurrently, cells were subjected to differentiation towards adipocytes and osteoblasts by using Chemicon MSC Adipogenesis kit (Millipore; USA) and Chemicon MSC Osteogenesis kit (Millipore), respectively. hWJMSCs were seeded at a density of 2× 10^4^ cells/cm^2^ and cells were directed to differentiate for 21 days in adipogenic differentiation medium. The presence of lipid vacuoles was confirmed by Oil Red O (Sigma-Aldrich, USA) staining. Meanwhile, osteogenic differentiation was carried out by culturing cells at a seeding concentration of 4× 10^4^ cells/cm^2^ under osteogenic differentiation medium for 21 days. Successful osteogenic differentiation was verified by Alizarin Red S (Sigma-Aldrich) staining. Cell nuclei were then counter-stained with hematoxylin.

### Preparation of Erythropoietin-Encoded Lentiviral Particles

The present study involved modification of MSCs with third generation self-inactivating (SIN) human immunodeficiency virus-1-based (HIV-1), *vsv-g* pseudotyped lentiviral vector, carrying human *EPO* and green fluorescent protein (GFP) genes. The pReceiver-Lv183 lentiviral transfer plasmid encoding for both human EPO (NCBI accession number: NM_000799.2) and *GFP* genes was purchased from GeneCopoeia (Rockville, MD, USA). The *EPO* gene was verified by reverse transcription-polymerase chain reaction (Supplementary Figure S1). The lentiviral plasmids were assembled in 50%–70% confluent human embryonic kidney 293FT cells (Invitrogen, USA) at 37°C in air with 5% CO_2_ for 8 h, using Endofectin lenti reagent (GeneCopoeia) to produce recombinant lentiviral particles. After replacement with fresh culture medium containing 1× TiterBoost reagent (GeneCopoeia), the transfected 293FT cells had grown to confluence and exhibited green fluorescence in their cytoplasm when examined under an inverted fluorescence microscope (Olympus, Japan) for green fluorescence (Supplementary Figure S2). Following 24, 48 and 60 h post-transfection, the harvested supernatants were pooled and filtered through a 0.22-μm filter prior to centrifuging at 500× *g*, 4°C for 8 min to remove cell debris. The culture supernatants from transfected cells were further concentrated using Amicon ultra centrifugal filter (Merck Millipore, Germany) for 90 min at 4000× *g*, 4°C. The concentrated lentiviral particles in the supernatants were used for transduction experiments.

### Transduction and Sorting of hWJMSCs

The human *EPO* gene was transduced into hWJMSCs (P3 to P6) by incubation with supernatants containing recombinant lentiviral particles, with 8 μg/ml polybrene supplement (Sigma-Aldrich). Following to 8 h of exposure, lentiviral particles were removed and replaced with MSC culture media. Transduced MSCs were culture-expanded and transduction efficiency was verified by detecting the GFP expression with fluorescence microscopy and flow cytometer. Following sub-culturing, the cells were further stained with CD44 (BD Biosciences) surface marker expression, raised from mouse against human epitopes, with APC fluorochrome, at 4°C for 30 min, in dark. In parallel, unstained, corresponded fluorochrome of non-specific isotype-labeled cells and non-transduced MSCs were used as controls. The stained samples were assessed using BD FACSAria III (BD Biosciences) and were gated from the control plots, in order to account for the autofluorescence of MSCs. Gating at FACS acquisition was applied to eliminate any cell death and cell debris. Ten thousand cells were acquired and the data from stained cells were acquired using FACSDiva 6.1.3 (BD Biosciences) software.

The GFP+ cell population was also sorted using the flow cytometer from the gated CD44+ cell population. The cell sorter was set up for aseptic sorting according to the manufacturer’s suggested guidelines and calibrated for sorting using Accudrop beads (BD Biosciences). The sorted cell population was aseptically maintained in DMEM/F-12 culture medium supplemented with 15% heat-inactivated FBS at a seeding density of 6000 cells/cm^2^ in a six-well culture dish. The sorted GFP-positive MSCs that constitutively expressed EPO were defined as MSC-EPO.

### Determination of EPO Expression by Enzyme-Linked Immunosorbent Assay (ELISA)

Sorted MSC-EPO were culture-expanded to 80% confluence before being replenished with DMEM/F-12 medium supplemented with 10% FBS for 72 h prior to harvesting the culture supernatant for further experimentation. Supernatants, termed MSC-EPO-conditioned medium (MSC-EPO-CM), were collected by centrifuging at 500× *g* for 8 min, to remove cellular debris, and purified through a syringe filter of 0.22 μm. The conditioned medium was used for the evaluation of EPO secretion and cytoprotective role in glutamate-induced cell death. In parallel, conditioned medium was collected from non-transduced MSCs, termed MSC-conditioned medium (MSC-CM) and served as control cultures.

Secreted EPO protein from culture supernatants were performed by ELISA (eBioscience, USA), in accordance to the manufacturer’s recommended protocol. Secreted proteins were measured using a microplate ELISA reader (Molecular Devices, USA) at 450 nm. Optical imperfections were corrected at 650 nm and the resulted optical density (OD) was used to subtract background control containing culture medium only. The OD values were used to estimate the EPO secretion according to the standard curve. The results representing three independent experiments (*n* = 8).

### Analysis of Mitochondrial Membrane Potential (ΔΨm)

Y79 cells were seeded at 5× 10^5^ cells/well in 6-well culture plate for 24 h prior to incubating with glutamate at IC50 of 67 mM glutamate solution (Supplementary Figure S3) in MSC culture medium, MSC-CM, and MSC-EPO-CM for 24 h. Following washing with PBS, the cells were incubated with JC-1 (5,5′,6,6-tetrachloro-1,1,3,3-tetraethylbenzimidazolylcarbocyanineiodide) dye (BD Biosciences) for 20 min. The stained cells were rinsed thrice with PBS to remove remnants of JC-1 before data acquisition. Ten thousand events from stained cells were acquired using a flow cytometer and FACSDiva software.

### Cytoprotective Effect of MSC-EPO-CM on Y79 Cells

The cytoprotective effect of MSC-EPO-CM was evaluated by culturing Y79 at 50,000 cells/well in 96-well culture dish for 24 h at 37°C in a 5% CO_2_ incubator. After 24 h, the cells were pre-disposed to 50 μl of MSC-CM and MSC-EPO-CM (EPO = 109.7 mI.U/ml) for 1 h, before being exposed to 50 μl glutamate solution (final concentration of 67 mM; Gibco) for 24 h. Twenty microliters of 300 μg/ml MTS (3-(4,5-dimethylthiazol-2-yl)-5-(3-carboxymethoxyphenyl)-2-(4-sulfophenyl)-2H-tetrazolium) solution (Promega) was added to 100 μl culture, for 3 h, at 37°C in 5% CO_2_. The quantity of formazan product formed at absorbance value of 490 nm was recorded by colorimetric microplate reader (Molecular Devices). The percentage of cell recovery was calculated by normalizing the mean absorbance value of the test group with the untreated control cells in absence of glutamate, multiplied by 100%.

### Statistical Analysis

The quantitative data were indicated as mean values ± standard error of the mean (SEM) of three independent set of experiments. The statistical analysis of EPO protein secretion was acquired using Prism 5.0 (GraphPad) with an unpaired, two-tailed *t*-test. Normality of data distribution was obtained using the D’Agostino and Pearson omnibus normality test with *P* < 0.0001 values were considered to be statistically significant. Statistical data analysis of cell viability was conducted by SPSS 21.0 (IBM Corporation, USA) software for the one-way Analysis Of Variance (ANOVA) and the *post hoc* Bonferroni’s multiple comparison tests. Normality of distribution and equal variance of the data were confirmed using the Kolmogorov-Smirnov with Lilliefors and Levene’s tests, respectively. *P* < 0.05 values considered to be statistically significant.

## Results

### Culture, Expansion and Characterization of hWJMSCs

**Figure 1:**
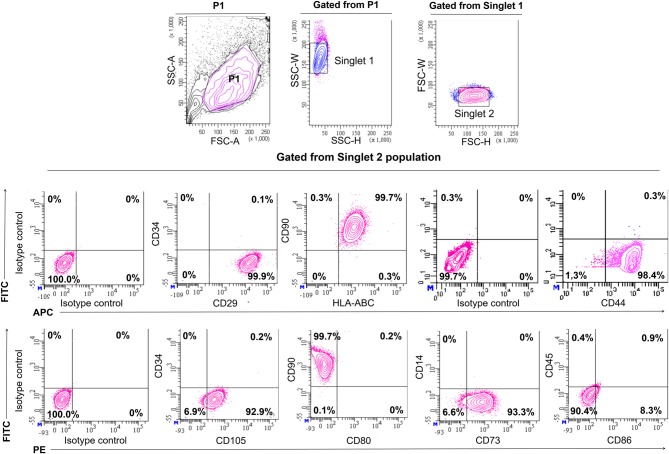
Immunophenotyping of human mesenchymal stem cells from Wharton’s jelly. Characterization of Mesenchymal Stem Cells (MSCs) for the expression of surface markers by flow cytometric analysis on the third passage. Cell population was gated as P1. Identification of singlet cells (singlet 1 and singlet 2) were used to remove cell clumps remaining after disaggregation and to eliminate autofluorescence events. The remaining events were considered to be singlet cells. Flow cytometry showed that gated cell populations from singlet 2 were positive for CD90, CD73, CD105, CD29, CD44 and HLA-ABC but did not express CD34, CD14, CD45, CD80 and CD86. For each antibody, isotype-matched mouse immunoglobulin γ antibody was used in unstained controls.

In order to confirm the characters of MSCs, the surface antigen phenotype of the culture-expanded cells was assessed by flow cytometry according to the recommendations by Dominici et al. (2006) which proposed on the minimal defining criteria for MSCs. Expanded cells at passages three to five expressed high levels of CD90 (99.7%), CD73 (93.3%), CD105 (92.9%), CD29 (99.9%), CD44 (99.1%) and HLA-ABC (99.7%), and were negative for the hematopoietic surface antigens of CD34, CD14, and CD45, and the co-stimulatory surface antigens, CD80. There was minute presence of co-stimulatory suface antigens of CD86 (8.3%), which is essential for stimulating T helper cell activity (Figure 1). All contour plots were compared to specific isotype controls, thus confirming that the cultured cells are homogenous. The results indicated that the isolated and culture-expanded cells showed immunophenotypic characters of MSCs.

**Figure 2:**
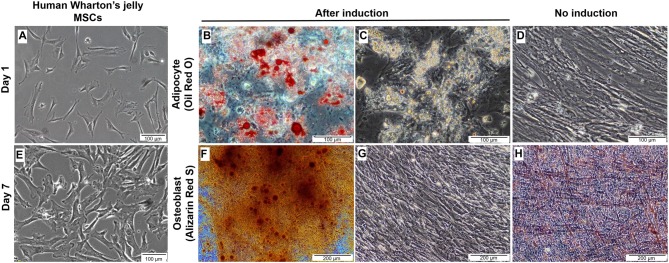
Culture, expansion and characterization of mesenchymal stem cells from human Wharton’s jelly. **(A)** Morphology of single cell-derived, clonally expanded MSCs. **(B)** Cultured MSCs were then culture-expanded and maintained between 80% and 90% confluence. **(C,D)** Differentiation of human Wharton’s jelly MSCs into adipocytes. The lipids were stained red with Oil Red O when compared to non-induced cells and cell nuclei were stained blue with hematoxylin **(C)**. Small lipid droplets were observed in the cytoplasm of the cells during early incubation and they became large due to lipid accumulation **(D)**. **(E)** The non-induced MSCs cultured in the growth media were stained negative by Oil Red O. **(F,G)** Morphological examination of MSCs induced towards osteogenic lineage. Small, crystal-like elements were observed accumulated across the monolayer cell surface at the end of the differentiation stage. The overcrowded crystals created difficulty in distinguishing the morphological changes in the cells **(G)**. The amorphous calcium deposit was stained orange red at pH 4.0 with Alizarin Red S, when compared to non-induced culture **(F)**. **(H)** The non-induced MSCs cultured in the growth media were stained negative by Alizarin Red S. Cells were imaged with a phase contrast microscope.

The majority of the cultured cells formed a monolayer of spindle-shaped cells 24 h after initial seeding (Figure 2A). Cell cultures at passages three to five were grown to 80%–90% confluence prior to adipogenic and osteogenic mesenchymal lineages induction (Figure 2B). During adipogenesis, induced cells were observed to undergo morphological changes displaying a more circular or cuboid shape. Lipid droplets were found in the cytoplasm of induced cells through the appearance of multiple tiny intra-cytoplasmic lipid droplets and were verified by positive Oil Red O staining (Figures 2C,D). These results were compared to control non-induced cells, which displayed no morphological changes, the absence of fat droplets, and negative staining with Oil Red O stain (Figure 2E). Meanwhile, during osteogenic differentiation, the cells exhibited close-distended cell bodies and stained positively by Alizarin Red S. This stain is useful to identify presence of calcium mineralization (Figure 2F). Calcium deposits were shown as amorphous accumulations between cells (Figure 2G). The non-induced cells were stained negative by Alizarin Red S (Figure 2H). These results indicated the MSCs showed successful differentiation into adipogenic and osteogenic lineages.

### Verification of Transduction Efficiency Based on EPO Secretion From EPO-Transduced MSCs (MSC-EPO) *in Vitro* by Enzyme-Linked Immunosorbent Assay (ELISA) and Flow Cytometry

**Figure 3:**
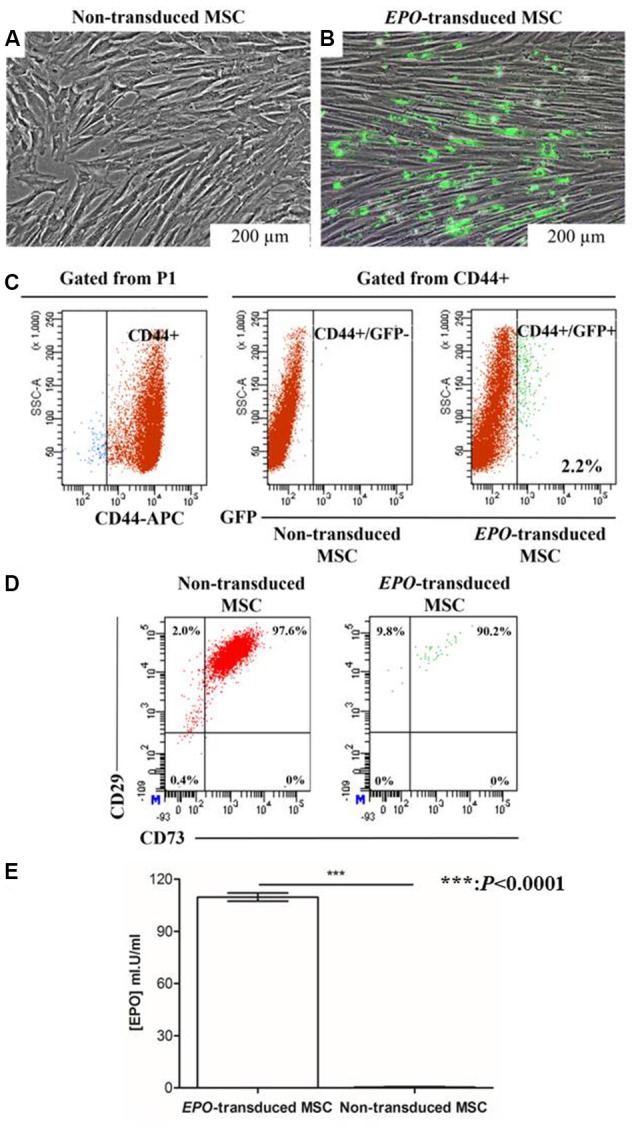
Determination of transduction efficiency based on green fluorescent protein (GFP) expression and erythropoietin (EPO) concentration in the supernatant of transduced cells. **(A)** Fluorescence microscopy images of transduced cells 21 days after transduction, and **(B)** non-transduced cells. **(C)** Flow cytometry dot plots showing the transduction efficiency of GFP positive non-transduced (red) and *EPO*-transduced (green) MSCs. The percentage of GFP+ MSCs upon transduction was measured by flow cytometry. Numbers indicate the percentage of cells in each gate. Fluorescence intensity of *EPO*-transduced MSCs were gated from the P1 population of non-transduced MSCs and referred to as GFP+. Flow cytometry showed that gated cell populations from CD44+ were positive for GFP. **(D)** Flow cytometry dot plots showed that transduced cell populations from GFP+ cells were positive for CD73 and CD29. An isotype control was included in the experiment to identify background fluorescence (data not shown). **(E)** EPO concentration in the conditioned medium of transduced MSCs. EPO concentration was significantly increased in the conditioned medium collected from transduced MSCs compared with that collected from non-transduced MSCs. Data are reported as the mean ± standard error of the mean (SEM) of three independent experiments (*n* = 8). *P < 0.0001 respect to non-transduced MSCs by unpaired two-tailed Student’s *t*-test.

Transduction in itself did not affect the morphological changes in the cell population. Transduced MSCs exhibited a typical elongated spindle-shaped after transduction over an extended cell culture (Figure 3A). The transduced cells showed green fluorescence when observed under the fluorescence microscope (Figure 3B). Lentiviral transduction of MSCs yielded approximately 2.2% of CD44+ cells expressing *EPO*-tagged GFP protein, with most of the successfully transduced cells expressing low fluorescence intensity of tagged protein, *GFP* (Figure 3C). Lentiviral transduction with the human *EPO* plasmid did not affect the phenotypic expression of CD73 and CD29 on the GFP+ sorted MSCs when comparing to non-transduced (GFP-) cells (Figure 3D).

The GFP-positive cells were then sorted by a flow cytometer to enhance transduced cell purity. Following sorting, the cells were culture-expanded and the supernatants were collected for EPO protein determination after 3 days. As shown in Figure 3E, EPO was secreted *in vitro* with a concentration as high as 109.7 ± 2.322 mI.U/ml (*n* = 8). EPO secretion was observed only in the supernatants from *EPO*-transduced MSCs, which indicates that transduction was successful and transduced MSCs were able to synthesize and process EPO appropriately. The standard curve for EPO determination was established using sandwich ELISA.

### MSC-EPO-CM Ameliorated Glutamate-Induced Neurotoxicity

In order to establish a suitable *in vitro* human retinal model for the evaluation of neuroprotective effect of MSC-EPO-CM in retinal degeneration, Y79 cells was used in the following study. Cellular recovery of human Y79 was evaluated after the addition of MSC-CM and MSC-EPO-CM at a concentration of glutamate that elicits neurotoxicity. The cell viability was quantified by using a colorimetric assay based on MTS reduction of viable cells into a colored formazan product. A dose-dependent declination in cell viability was distinguished after exposing the cultured cells to glutamate (GA) for 24 h, as assessed by the MTS assay, with an estimated IC50 of 67.42 ± 0.306 mM (Supplementary Figure S3).

**Figure 4:**
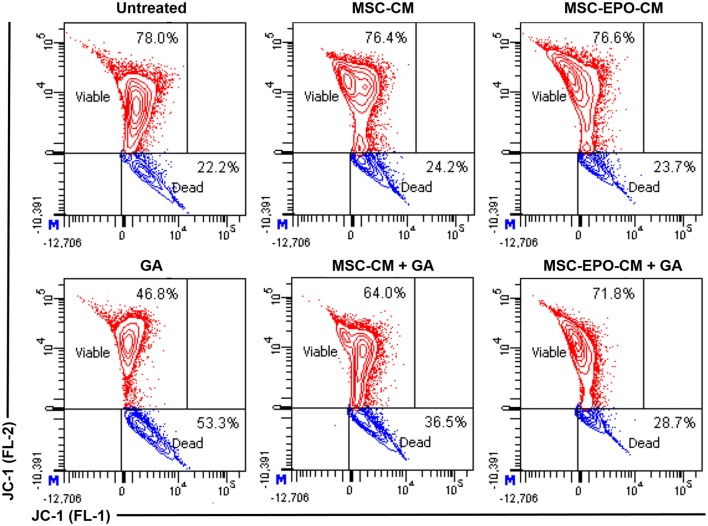
Changes in the mitochondrial membrane potential (ΔΨm) of glutamate-induced Y79 cells. (Upper panel) The status of the ΔΨm in the Y79 cells in the absence and (lower panel) presence of 67 mM glutamate was examined at 24 h after co-treatment with conditioned medium derived from non-transduced (MSC-CM) and *EPO*-transduced MSCs (MSC-EPO-CM). Y79 cells exposed to the same concentration of glutamate solution in fresh MSCs culture medium was used as a control. JC-1 is a selective membrane dye that enters the negatively charged mitochondrial matrix and was used to measure changes in the ΔΨm. In a living cell, healthy mitochondria was polarized and JC-1 was rapidly taken up by such mitochondria. The increase uptake could lead to the aggregation of JC-1, which demonstrated red fluorescence when measured in the FL-2 channel of a flow cytometer. On the other hand, JC-1 did not accumulate in a dying cell and formed monomer. The monomers showed lower fluorescence in the FL-2 and increased fluorescence in the FL-1 channel. The flow cytometric analysis was shown in the contour plots.

As shown in Figure 4 (upper panel), untreated Y79 cells displayed a distinct fluorescence in the FL-2 channel with 78.0% indicating viable cells population. Despite exposing the cells to MSC-CM and MSC-EPO-CM for 24 h, there were no significant differences in the FL-2 fluorescence intensity with viable cells population of 76.4% and 76.6% to that of untreated Y79 cells, respectively. However, incubation with 67 mM GA displayed a substantial reduction in cell survivability to 46.8%, reflecting a loss of ΔΨm and cell death (lower panel). Further flow cytometric analysis revealed that co-treating cells with MSC-CM and MSC-EPO-CM in the presence of GA lead to an increase of viable cells population from 46.8% to 64.0% (MSC-CM + GA) and 71.8% (MSC-EPO-CM + GA), respectively. In addition, we also evinced a more pronounced cell population in the FL-2 channel when the cells were co-treated with MSC-EPO-CM (MSC-EPO-CM + GA) compared with the MSC-CM (MSC-CM + GA) group, which reflects greater alleviation in the loss of ΔΨm. These results confirmed the protective effect of combined treatment of MSC-EPO-CM in preventing mitochondrial dysfunction associated with mitochondria-dependent apoptotic pathway.

**Figure 5:**
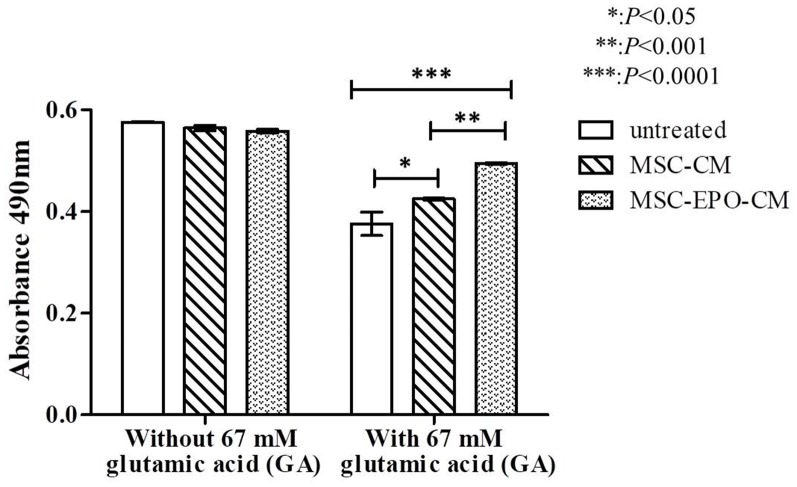
*In vitro* effect of glutamate-induced toxicity on Y79 cell viability in the presence of conditioned media. Recovery of 67 mM glutamate-induced Y79 cells treated with conditioning medium derived from non-transduced (MSC-CM) and *EPO*-transduced (MSC-EPO-CM) MSCs was analyzed by one-way analysis of variance (ANOVA) followed by Bonferroni’s test for each mean comparison. *P < 0.05 values were considered to be statistically significant. Results were expressed as mean ± standard error of the mean (SEM) of three independent experiments (*n* = 8). Y79 cells exposed to the same concentration of glutamate solution in fresh MSCs culture medium was used as a control.

Further study indicated that the viability of Y79 incubated with MSCs and MSC-EPO conditioned medium was similar to that of cells incubated in fresh culture medium without addition of glutamate. The addition of 67 mM of glutamate, co-treatment of MSC-CM or MSC-EPO-CM increased the cell viability from 65.27 ± 0.022% to 73.77 ± 0.002% (*P* = 0.032, *n* = 8) or 85.88 ± 0.001% (*P* = 0.0001, *n* = 8), respectively (Figure 5). The increase in the cell viability suggested a greater neuroprotective effect of MSC-EPO conditioned medium, compared with MSC conditioned medium alone, on glutamate-induced retinal neurotoxicity.

## Discussion

In this study, we transduced EPO gene into human Wharton’s Jelly derived MSCs and evaluated the rescue potential of secreted EPO following glutamate-induced cell death in Y79 cell line. The flow cytometric results indicated that 2.2% of MSCs were successfully transduced with *EPO*-tagged *GFP* gene (Figure 4) and these cells co-expressed for MSC surface marker CD44. A study previously reported that positive expression of CD44 in MSC culture indicates a well-preservation of MSC stemness (Zhu et al., 2006; Maleki et al., 2014). Hence, our study suggested that the transduction of MSCs did not compromise MSC stemness properties. Current study was established using pre-treatment approach to demonstrate the anti-oxidative defense system of MSC coupled with EPO protein against glutamate-induced oxidative damage. This approach provides a proper understanding of the treatment efficacy in circumventing the low survival ratio of Y79 cells under harsh oxidative stress and inflammatory environment and allows an effective way to postulate the beneficial effect of MSC-EPO under simulated retinal degenerative conditions.

Here, we established an *in vitro* model of oxidative stress-induced retinal cell by using glutamate and aim to validate the retinal cell survivability in the presence of MSC-EPO. The Y79 cell line is a human retinoblastoma cell expressing a heterogeneous populations for both immature and mature retinal cell type-specific markers, such as Cone Rod Homeobox (CRX), Visual System Homeobox 2 (VSX2), Protein Kinase C-alpha (PKC-α), Retinoid X Receptor gamma (RXRγ), thyroid hormone receptor beta-2 (TRβ-2), Neural Retina-Specific Leucine zipper (NRL) and recoverin (Sakata and Yanagi, 2008; Xu et al., 2009; Oshikawa et al., 2011; Cassidy et al., 2012; Han and Townes-Anderson, 2012). Hence, it is a suitable *in vitro* model to represent the responses of a broader range of human retinal neuronal cells upon exposure to glutamate. It is worth noting that exposure to high level of glutamate has been implicated to result in mitochondrial dysfunction in AMD, retinitis pigmentosa, and diabetic retinopathy, represented by the reduction in its membrane potential. Following that, there is a massive release of cytochrome c and numerous apoptotic proteins, such as pro-caspase 9, which will ultimately lead to cell destruction (Tait and Green, 2008). We found that co-treatment with MSC-EPO has the ability to attenuate apoptotic cell death by restoring the mitochondrial membrane potential via inhibition in the mitochondria-dependent apoptosis pathway.

The current results are dependent on the amount of EPO secretion, influenced by transduction and gene expression. Hence, before the extension to the *in vivo* studies to further verify the MSC-EPO-mediated neuroprotective activity in a retinal disease animal model, it is vital to establish a stable clone of transduced MSC-EPO and determine the minimal amount of EPO sufficient to exert the neuroprotective action. In clinical settings, patients will normally have experienced substantial amount of oxidative-induced stress, and delivery of MSC-EPO may not be beneficial to reverse the damage incurred by the stress. However, early treatment with transplantation of MSC-EPO may be helpful to prevent from further damage by rescue of cell death and new cell regeneration.

Based on our findings, we hypothesized that MSCs expressing EPO (MSC-EPO) can rescue retinal cells from cell death through a number of mechanisms, such as the protective effect of trophic factors secreted by MSCs on retinal cells as well as dual protective mechanisms of EPO released from transduced MSCs on retinal cells. In contrast to the direct use of MSCs, Roth et al. (2016) have shown that supernatants collected from MSC cultures were able to restore retinal architecture and function response in the ischemia-reperfusion injury rat model. Cell recovery was postulated to be associated with anti-apoptotic and anti-inflammatory associated growth factors [tumor necrosis factor (TNF), vascular endothelial growth factor (VEGF)-A, NGF and Granulocyte Macrophage-Colony Stimulating Factor (GM-CSF)] and cytokines of interleukin family (Roth et al., 2016). Likewise, MSCs were also shown to, following corneal injury, promote corneal epithelium regeneration and, simultaneously, attenuate corneal opacity, neovascularization, and to increase the expression level in a wide range of pro-inflammatory cytokines, including Alpha-Smooth Muscle Actin (SMA), inducible Nitric Oxide Synthase (iNOS), matrix metalloproteinase (MMP)-9, transforming growth factor (TGF)-β1 and VEGF, in comparison to injured eyes (Cejka et al., 2016).

Additionally, ample studies have shown that intravitreal transplantation of MSCs expressing BDNF could significantly restore the structural and functional integrity of the retina with an increase of intraocular pressure (Harper et al., 2011). Nevertheless, Ola et al. (2013) reported a diminished neurotrophin BDNF receptor (tropomyosin-related kinase B; TrkB) expression on the retinal tissue of diabetic rats, and thus would limit the therapeutic proteins from achieving greater and direct protection against degenerating cells (Ola et al., 2013; Perígolo-Vicente et al., 2013). Further study has shown that delivery of EPO in the vitreous cavity of diabetic rat model could enhance the expression of both BDNF and TrkB (Wang and Xia, 2015). Hence, the delivery of EPO into MSCs might be useful to overcome the treatment efficiency with stem cells transplantation.

Numerous studies have indicated that the administration of EPO prior to stem cell transplantation may regulate the microenvironment and enhance stem cell survivability (Zhang et al., 2008; Chung et al., 2009; Liu et al., 2013; Busch et al., 2014), and increase tissue repair, even when low numbers of cells are transplanted. In our previous review, the mechanisms of protection by EPO, such as anti-apoptosis, anti-inflammatory, anti-oxidative and neuroregeneration capabilities, were found to be mediated through the modulation of the mitogen-activated protein kinase (MAPK), signal transducer activator-of-transcription (STAT), phosphatidylinositol-3-kinase/Akt (PI3-K/Akt), and nuclear factor-kappa light chain enhancer-of-activated B cells (NF-κB) signaling cascades (Shirley Ding et al., 2016). In addition to the widely distributed occurrence of EPO and its associated EPO receptors (EPOR) in retinal tissue (Shirley Ding et al., 2016), as well as the ability of EPO to cross the BRB, studies found that EPO reacts with the damaged photoreceptors by crosslinking with the EPOR site (Grimm et al., 2002). Furthermore, accumulating findings have also outlined the promising use of EPO (Zhu et al., 2009; Mok et al., 2012; Boesch et al., 2014) in the treatment of retinal degenerative diseases, such as AMD (Wang et al., 2009), retinitis pigmentosa, and diabetic retinopathy (McVicar et al., 2011). Coupling EPO with MSC therapy also increases the probability of successful MSC transplantation in a harsh microenvironment, which will lead to amplifying the regenerative potential of MSCs.

Despite the successful transduction and expression of EPO in the cells, the present study has utilized third generation, SIN vectors for the production of MSC-expressing EPO through a single transduction procedure, thus greatly reducing the risk of proto-oncogene activation on the neighboring cells. Nevertheless, the risk of insertional oncogenesis following lentiviral vector integrations has been reported. Furthermore, there are concerns that the constant activation of EPO expression may evoke side effects involving neovascularization (Xiong et al., 2009; Mohan et al., 2012), therefore, a well-regulated EPO secretion with the addition of tetracycline could be the ideal approach to limit irreversible elevation of EPO secretion in the eye.

## Conclusion

In conclusion, we were able to transduce human mesenchymal stem cells derived from Wharton’s jelly with lentiviral encoding for *EPO* gene. The secreted EPO protein could enhance retinal neuron cell survivability following induction with glutamate. Taken together, the current study provides a rational strategy whereby MSCs could serve as a candidate for the delivery of *EPO* therapeutic gene in the treatment of retinal degenerations.

## Author Contributions

PLM conceived the experimental study design, analyzed the data and edited the manuscript. SD conducted the experiments, composed this manuscript, prepared the figures and was responsible for statistical elaboration of the data. SKS supported with study design, analyzed and commented on both data and figures. MSAK analyzed and edited the manuscript. All authors reviewed the manuscript.

## Conflict of Interest Statement

The authors declare that the research was conducted in the absence of any commercial or financial relationships that could be construed as a potential conflict of interest.

## Supplementary Material

FIGURE S1 Schematic representation of the third-generation system comprising the packaging mix and *EPO*-encoding lentiviral transfer vector (Ex-A1011-Lv183) constructs. **(A)** Vector map diagram for pReceiver-Lv183 lentiviral transfer plasmid encoding human erythropoietin (*EPO*) and enhanced gene-tagged green fluorescent protein (*GFP*). The self-inactivation of HIV-based lentiviral vector (Ex-A1011-Lv183) is a third-generation lentiviral vector containing the cis-active sequences of HIV-1 that are required for encapsidation of the viral vector genome and for viral transduction of target cells, as well as the C-terminal tagged GFP expression under the control of human cytomegalovirus (CMV) promoter. The upstream 5′ LTR contains the *rsv* promoter to drive the production and expression of viral RNA in the packaging cells. The U3 enhancer sequences in the region of the 3′ LTR are deleted to ensure self-inactivation of the lentiviral construct after transduction and integration into the genomic DNA of the target cells. **(B)** The HIV packaging mix contains two packaging plasmids, one encoding *gag-pol*, another encoding *rev*, and an envelope protein (Env) encodes *vsv-g*, to allow extensive infectivity. **(C)** Confirmation of *EPO* gene from pReceiver-Lv183 plasmid. Resulting plasmid DNA was electrophoresed on an agarose gel in the following lanes: lane 1 (100 base pairs DNA ladder), lane 2 (positive control containing 129 ng/μl of *EPO*-encoding lentiviral plasmid), lane 3 (negative control containing no *EPO*-encoding lentiviral plasmid), and lane 4 (containing 129 ng/μl of transformed *EPO*-encoding lentiviral plasmid). Bands were of the expected size, as shown in the schematic of the PCR products.

FIGURE S2 Lentiviral transfection of the human kidney (293FT) cell line with human erythropoietin (*EPO*) tagged with enhanced green fluorescent protein (*GFP*) at 12 h, 24 h, 48 h and 60 h post-transfection. **(A)** Phase contrast image of a healthy culture of 293FT cell line derived from primary embryonic human kidney cells. On day 7, the adherent cells were at 60%–70% cell confluence and had the appearance of epithelial-like cells in polygonal-shapes. **(B–D)** Morphological change in cultured cells after 12 h post-transfection with *EPO*-encoding lentiviral plasmid. **(E–M)** At 24 h, 48 h and 60 h, the appearance of large, multinucleated syncytia was observed across the cell culture. Expression of GFP was visualized using inverted fluorescence microscopy, indicating efficiency of transfection. Cells were imaged by phase contrast microscopy and inverted fluorescence microscopy.

FIGURE S3 Dose response curve of glutamate on Y79 retinal cell. A dose responsive assay on Y79 cells upon exposure to increasing concentrations of glutamate, GA (0–100 mM) for 24 h. The dose responsive effect of GA on Y79 retinal cell was applied as IC_50_ value that representing the concentration of glutamate that is required to inhibit 50% of cell growth, in this experiment. Determination of the IC_50_ was calculated by using the best-fit curve model analyzed with GraphPad Prism software. IC_50_ of glutamate was estimated at 67.42 ± 0.31 mM (mean ± SEM; *n* = 9).
