# Supplementary figures and images for: Human Mesenchymal Stem Cells Expressing Erythropoietin Enhance Survivability of Retinal Neurons Against Oxidative Stress: An In Vitro Study
Source: Front Cell Neurosci. 2018 Jul 31;12:190. doi: 10.3389/fncel.2018.00190 (PMC6079241; doi:10.3389/fncel.2018.00190)

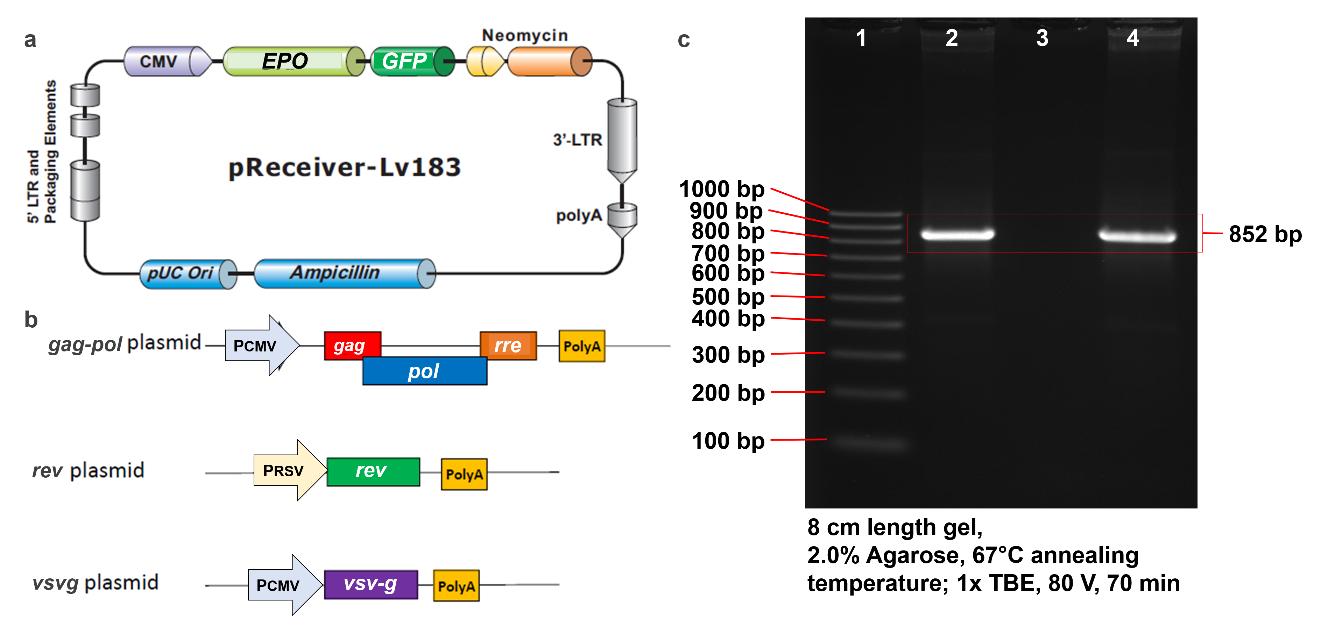

Supplement: FIGURE S1 — Schematic representation of the third-generation system comprising the packaging mix and EPO-encoding lentiviral transfer vector (Ex-A1011-Lv183) constructs. (A) Vector map diagram for pReceiver-Lv183 lentiviral transfer plasmid encoding human erythropoietin (EPO) and enhanced gene-tagged green fluorescent protein (GFP). The self-inactivation of HIV-based lentiviral vector (Ex-A1011-Lv183) is a third-generation lentiviral vector containing the cis-active sequences of HIV-1 that are required for encapsidation of the viral vector genome and for viral transduction of target cells, as well as the C-terminal tagged GFP expression under the control of human cytomegalovirus (CMV) promoter. The upstream 5′ LTR contains the rsv promoter to drive the production and expression of viral RNA in the packaging cells. The U3 enhancer sequences in the region of the 3′ LTR are deleted to ensure self-inactivation of the lentiviral construct after transduction and integration into the genomic DNA of the target cells. (B) The HIV packaging mix contains two packaging plasmids, one encoding gag-pol, another encoding rev, and an envelope protein (Env) encodes vsv-g, to allow extensive infectivity. (C) Confirmation of EPO gene from pReceiver-Lv183 plasmid. Resulting plasmid DNA was electrophoresed on an agarose gel in the following lanes: lane 1 (100 base pairs DNA ladder), lane 2 (positive control containing 129 ng/μl of EPO-encoding lentiviral plasmid), lane 3 (negative control containing no EPO-encoding lentiviral plasmid), and lane 4 (containing 129 ng/μl of transformed EPO-encoding lentiviral plasmid). Bands were of the expected size, as shown in the schematic of the PCR products. [file Image_1.TIF]

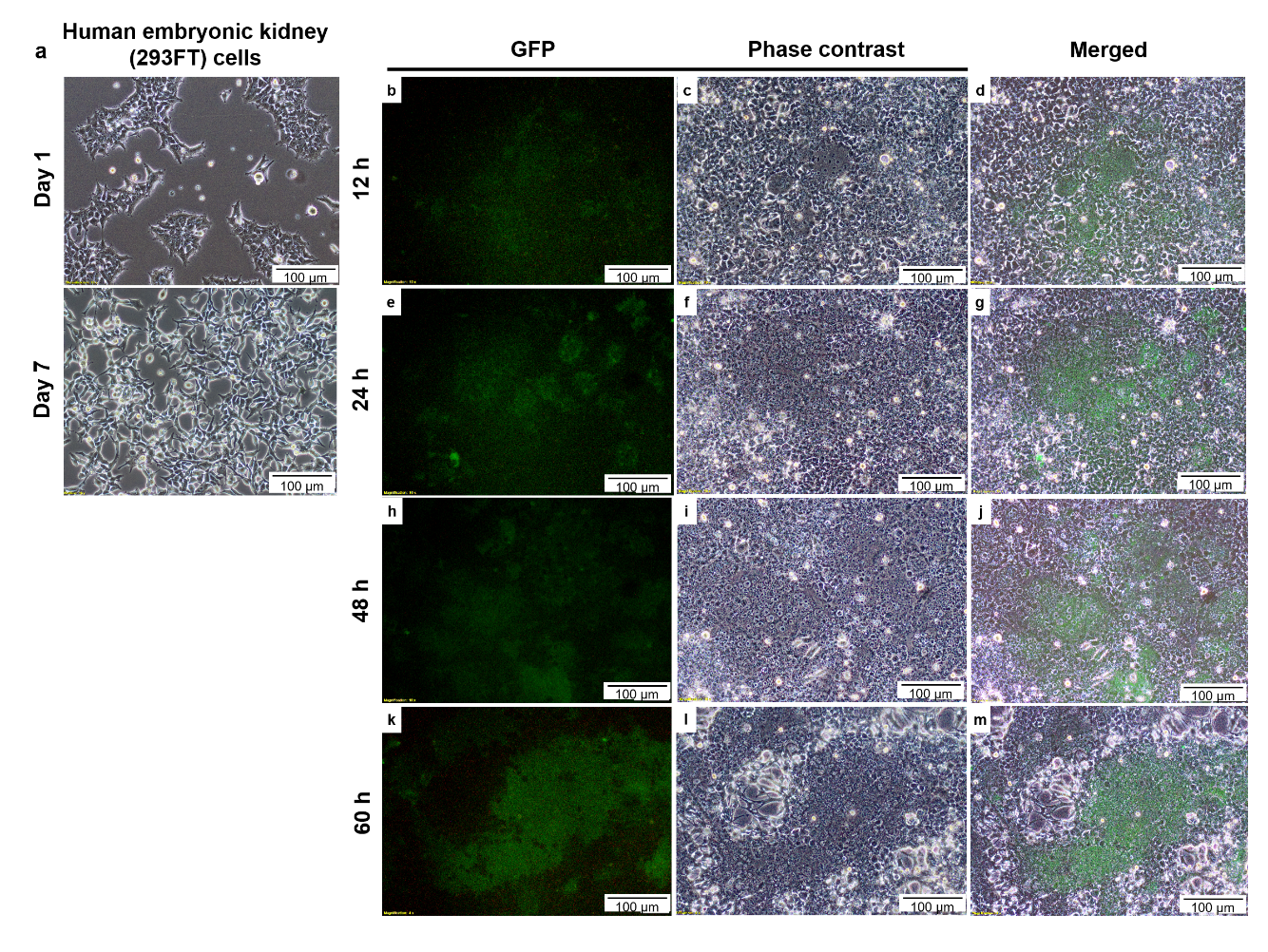

Supplement: FIGURE S2 — Lentiviral transfection of the human kidney (293FT) cell line with human erythropoietin (EPO) tagged with enhanced green fluorescent protein (GFP) at 12 h, 24 h, 48 h and 60 h post-transfection. (A) Phase contrast image of a healthy culture of 293FT cell line derived from primary embryonic human kidney cells. On day 7, the adherent cells were at 60%–70% cell confluence and had the appearance of epithelial-like cells in polygonal-shapes. (B–D) Morphological change in cultured cells after 12 h post-transfection with EPO-encoding lentiviral plasmid. (E–M) At 24 h, 48 h and 60 h, the appearance of large, multinucleated syncytia was observed across the cell culture. Expression of GFP was visualized using inverted fluorescence microscopy, indicating efficiency of transfection. Cells were imaged by phase contrast microscopy and inverted fluorescence microscopy. [file Image_2.TIF]

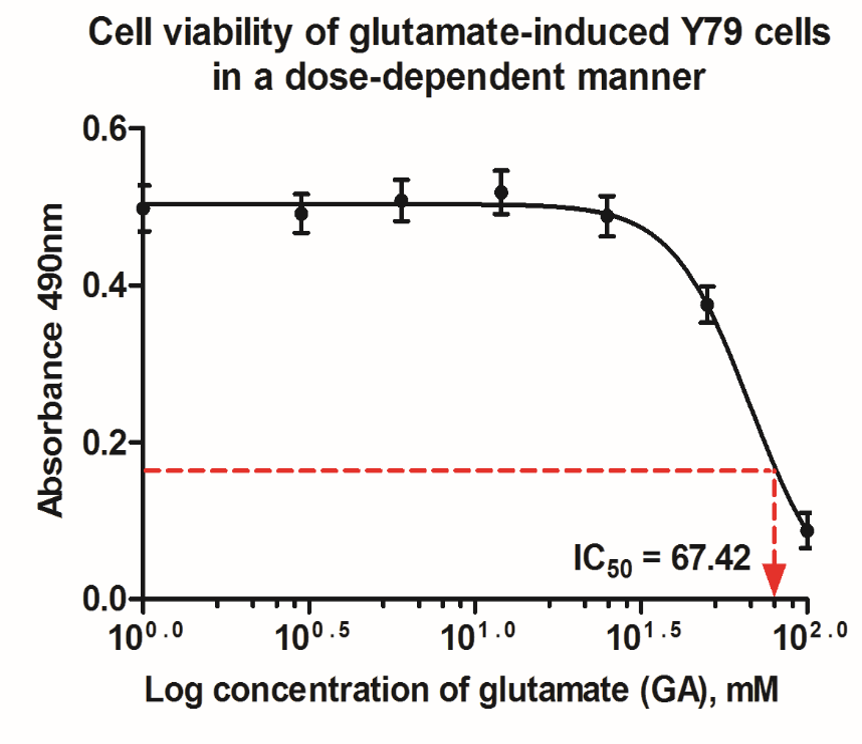

Supplement: FIGURE S3 — Dose response curve of glutamate on Y79 retinal cell. A dose responsive assay on Y79 cells upon exposure to increasing concentrations of glutamate, GA (0–100 mM) for 24 h. The dose responsive effect of GA on Y79 retinal cell was applied as IC50 value that representing the concentration of glutamate that is required to inhibit 50% of cell growth, in this experiment. Determination of the IC50 was calculated by using the best-fit curve model analyzed with GraphPad Prism software. IC50 of glutamate was estimated at 67.42 ± 0.31 mM (mean ± SEM; n = 9). [file Image_3.TIF]
